# Conformational ensemble of native α-synuclein in solution as determined by short-distance crosslinking constraint-guided discrete molecular dynamics simulations

**DOI:** 10.1371/journal.pcbi.1006859

**Published:** 2019-03-27

**Authors:** Nicholas I. Brodie, Konstantin I. Popov, Evgeniy V. Petrotchenko, Nikolay V. Dokholyan, Christoph H. Borchers

**Affiliations:** 1 University of Victoria -Genome British Columbia Proteomics Centre, Vancouver Island Technology Park, Victoria, British Columbia, Canada; 2 Department of Biochemistry and Biophysics, University of North Carolina, Chapel Hill, North Carolina, United States of America; 3 Segal Cancer Proteomics Centre, Lady Davis Institute, Jewish General Hospital, McGill University, Quebec, Canada; 4 Departments of Pharmacology, and Biochemistry and Molecular Biology, Pennsylvania State College of Medicine, Hershey, Pennsylvania, United States of America; 5 Department of Biochemistry and Microbiology, University of Victoria, Victoria, British Columbia, Canada; 6 Gerald Bronfman Department of Oncology, Jewish General Hospital, McGill University, Montreal, Quebec, Canada; Fudan University, CHINA

## Abstract

Combining structural proteomics experimental data with computational methods is a powerful tool for protein structure prediction. Here, we apply a recently-developed approach for *de novo* protein structure determination based on the incorporation of short-distance crosslinking data as constraints in discrete molecular dynamics simulations (CL-DMD) for the determination of conformational ensemble of the intrinsically disordered protein α-synuclein in the solution. The predicted structures were in agreement with hydrogen-deuterium exchange, circular dichroism, surface modification, and long-distance crosslinking data. We found that α-synuclein is present in solution as an ensemble of rather compact globular conformations with distinct topology and inter-residue contacts, which is well-represented by movements of the large loops and formation of few transient secondary structure elements. Non-amyloid component and C-terminal regions were consistently found to contain β-structure elements and hairpins.

## Author summary

As the population ages, neurodegenerative diseases such as Parkinson’s disease will become an increasing problem in many countries. Aggregation of the protein α-synuclein is the primary cause of Parkinson’s disease, but there is still a dearth of structural information pertaining to the native, non-aggregating form of this protein. A better understanding the structural state of the native protein may prove useful for the design of new therapeutics to combat this disease. In order to obtain more structural information on this protein, we have recently modelled the native α-synuclein protein. These models were generated using a novel approach which combines protein crosslinking and discrete molecular dynamics simulations. We have found that the α-synuclein protein can adopt several shapes, all with a similar topology, resembling a three fingered closed claw. A region of the protein important for aggregation was found to be protected from the surrounding biological environment in these conformations, and the stabilization of these structures may be a fruitful avenue for future drug research into mitigating the cause and effect of Parkinson’s disease.

## Introduction

α-Synuclein is involved in the pathogenesis of misfolding-related neurodegenerative diseases, in particular Parkinson’s disease [1, 2]. A misfolding event leads to the formation of oligomers which are believed to result in cell toxicity and which eventually lead to the death of neuronal cells [3]. α-Synuclein is thought to interact with lipid vesicles *in vivo* [4] and the toxicity is thought to be mediated via membrane disruption by misfolded oligomers [5]. Moreover, a prion-like spread of the pathology *via* the conversion of native α-synuclein molecules by toxic oligomers has been suggested [6]. Native α-synuclein is considered to be an intrinsically disordered protein, although there is evidence that some globular structure exists in solution, which may serve as a basis for understanding the mis-folding and oligomerization pathways. A number of biophysical methods, such as NMR, EPR, FRET, and SAXS—in combination with computational methods—have been applied to the study of intrinsically disordered proteins, including the structure of α-synuclein in solution [7–10]. In all of these cases, even a limited amount of experimental structural data was helpful in the characterization of the conformational ensemble of α-synuclein in solution.

Recently, we developed a method for determination of protein structures, termed short-distance crosslinking constraint-guided discrete molecular dynamics simulations (CL-DMD), where the folding process is influenced by short-distance experimental constraints which are incorporated into the DMD force field [11]. Adding constraints to DMD simulations results in a reduction of the possible conformational space and allows the software to achieve protein folding on a practical time scale. We have tested this approach on well-structured proteins including myoglobin and FKBP and have observed clear separation of low-energy clusters and a narrow distribution of structures within the clusters. The conformational flexibility of intrinsically disordered proteins, such as α-synuclein, brings additional challenges to the computational process [12]. In cases like this, proteins exist as a collection of inter-converting conformational states, and crosslinking data represents multiple conformations of a protein rather than a single structure. In addition, recent research indicates that traditional force fields with their parametrization are not ideal for providing an accurate description of disordered proteins, and tend to produce more compact structures [13]. Recently research has been focused on improving traditional state-of-the-art force fields and their ability to predict structures of disordered proteins without losing their accuracy for structured proteins [14]. In this work we use a Medusa force field [15–17] that is utilized in DMD simulations is discretized to mimic continuous potentials. DMD uses a united atom representation for the protein where all heavy atoms and polar hydrogens are explicitly accounted. The solvation energy is described in terms of the discretized Lazaridis-Karplus implicit solvation model [18] and inter-atomic interactions, such van der Waals and electrostatics, are approximated by a series of multistep square-well potentials.

Other additional potentials, such as pair-wise distance constraints [19, 20] and solvent accessibility information [21, 22] can also be readily integrated. During CL-DMD simulations there are no continuous forces that would drive the atoms to satisfy all constraints, rather generating conformational ensembles, which satisfy an optimal number of the constraints are generated. This, to some degree, naturally resolves conflicting experimental constraints. Thus, CL-DMD simulations are a viable computational platform for the structural analysis of intrinsically disordered proteins [23] in general, and α-synuclein in particular.

Here, we used the CL-DMD approach [11] to determine conformational ensembles of the α-synuclein protein in solution. During this process, α-synuclein was crosslinked with a panel of short-range crosslinkers, crosslinked proteins were enzymatically digested, crosslinked residues were determined by LC-MS/MS analysis, and the resulting data on inter-residues distances were introduced into DMD force field as external constraints. To experimentally validate the predicted structures, we analyzed α-synuclein using surface modification (SM), circular dichroism (CD), hydrogen-deuterium exchange (HDX), and long-distance crosslinking (LD-CL).

## Methods

All materials were from Sigma-Aldrich, unless noted otherwise.

### Structural proteomics

**Fig 1:**
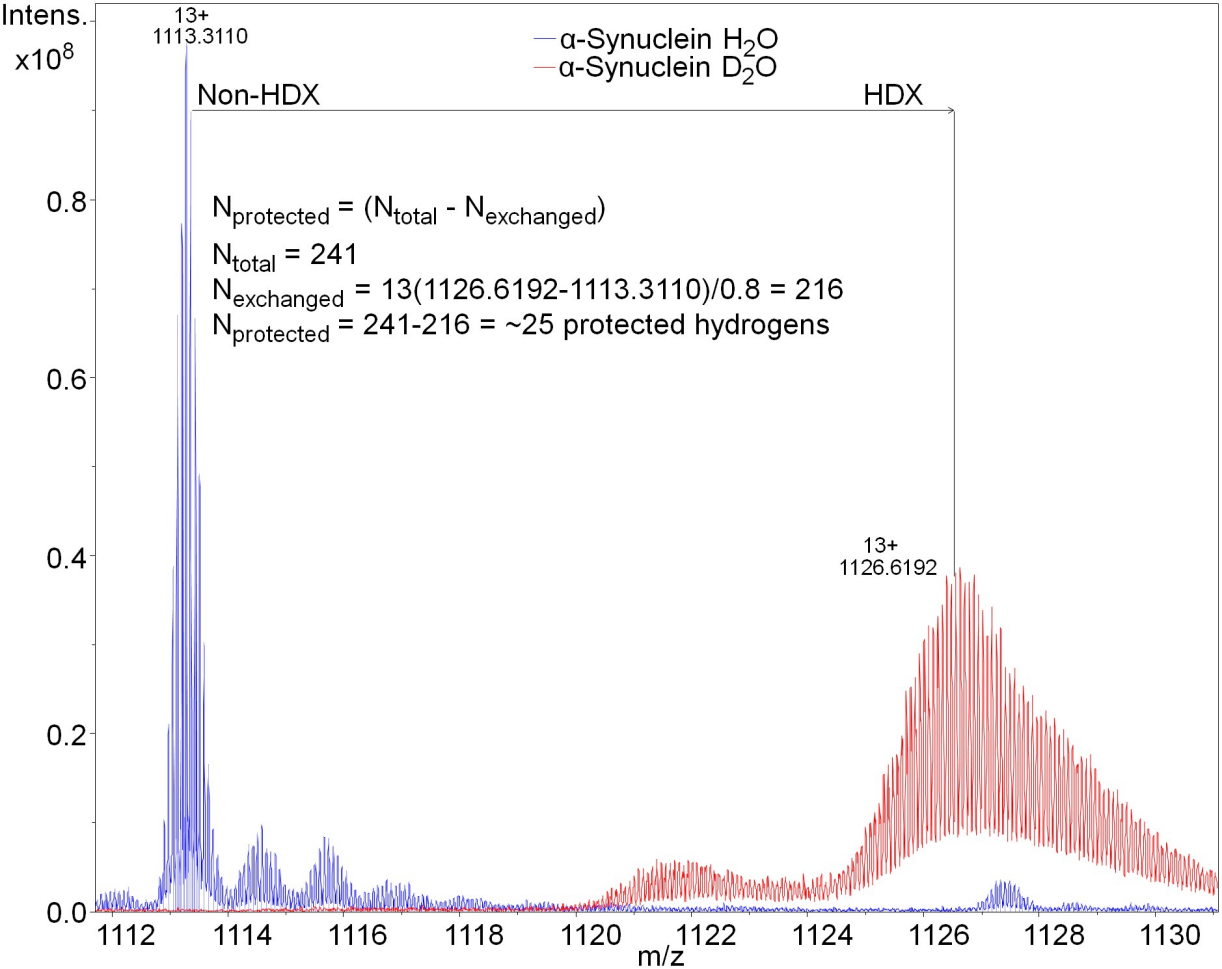
Hydrogen-deuterium exchange of α-synuclein. The total number of protected hydrogens was measured to be approximately 25 at 2s of exchange, indicating the existence of transient secondary structure.

α-Synuclein was crosslinked with a panel of short-range reagents azido-benzoic acid succinimide (ABAS-^12^C_6_/^13^C_6_), succinimidyl 4,4'-azipentanoate (SDA), [24] triazidotriazirine (TATA-^12^C_3_/^13^C_3_), [25] and 1-ethyl-3-(3-dimethylaminopropyl)carbodiimide (EDC) [26]. ABAS and SDA are hetero-bifunctional amino group-reactive and photo-reactive reagents, TATA is a homo-bifunctional photo-reactive reagent, and EDC is a zero-length carboxyl-to-amino group crosslinker. Crosslinked proteins were digested with proteinase K or trypsin proteolytic enzymes, and the digest was analyzed by LC-MS/MS to identify crosslinked peptides (S1 Table). We used an equimolar mixture of ^14^N- and ^15^N-metabolically labeled α-synuclein to exclude potential inter-protein crosslinks from the analysis and to facilitate the assignment of crosslinked residues based on the number of nitrogen atoms in the crosslinked peptides and the MS/MS fragments [27]. The distances between crosslinked residues are based on the length of the crosslinker reagents, and were introduced as constraints into the DMD potentials (see section below and [11] for additional details). A total of 30 crosslinking constraints were used in these DMD simulations (S1 Table). In addition, α-synuclein was characterized by top-down ECD- and UVPD-FTMS HDX and CD to determine the secondary-structure content (Fig 1 and S1 Fig). Quantitative differential surface modification experiments were performed with and without 8 M urea to determine the characteristics of the residues as exposed or buried (S2 Table). LD-CL was used to estimate the overall protein topology (S3 Table).

### Expression and purification of α-synuclein

α-Synuclein was expressed using a pET21a vector provided by Dr. Carol Ladner of the University of Alberta. The protein was expressed in E. coli BL21(DE3) bacteria and was purified as in [25]. Briefly, the protein was overexpressed with 1 mM IPTG in 1L LB cultures of BL21DE3 E. coli for 4 hours at 30°C. Cells were lysed with a French press and the lysate was heated at 70°C for 10 minutes and then centrifuged at 14000 g for 30 minutes. The soluble fraction was precipitated for 1 hour in 2.1 M (NH_4_)_2_SO_4_. α-Synuclein was then purified by fast protein liquid chromatography on a Mono Q 4.6/100 SAX column (GE Life Science), using a gradient from 50–500 mM NaCl, in 50 mM Tris at pH 8.0. Elution fractions containing α-synuclein were further purified by size exclusion on a Superdex 200 30/100 GL column (GE Life Science). For the expression of metabolically labeled ^15^N α-synuclein, 1L of M9 Minimal media was prepared with 1 g/L ^15^NH_4_Cl (Cambridge Isotopes) as the sole source of nitrogen. BL21(DE3) cells were grown overnight in 50mL of this media, then seeded into 1 L, grown to an A_600_ of approximately 0.8, and induced using 1 mM IPTG. After expression overnight at 30°C, ^15^N α-synuclein was purified as described above.

### Crosslinking

Unlabeled and ^15^N metabolically-labeled α-synuclein were mixed in a 1:1 ratio at a concentration of 20 μM in 50 mM Na_2_HPO_4_ and incubated overnight at room temperature prior to crosslinking. α-synuclein aliquots of 38 μL were then crosslinked using either 1 mM of the ABAS-^12^C_6_/^13^C_6_ crosslinker (Creative Molecules) or 30 mM of the EDC crosslinker. ABAS crosslinking reaction mixtures were incubated for 10 minutes in the dark to allow the NHS-ester reaction to take place, followed by 10 minutes of UV irradiation under a 25 W UV lamp (Model UVGL-58 Mineralight lamp, UVG) with a 254 nm wavelength filter. ABAS reaction mixtures were quenched with 10 mM ammonia bicarbonate. EDC reaction mixtures were incubated for 20 minutes. A portion of each crosslinking reaction mixture was checked by SDS-PAGE gel to see the extent of potential intermolecular crosslinked products. Aliquots were subsequently split and digested with either trypsin or proteinase K at an enzyme: protein ratio of 1:10. Digestion was quenched using a final concentration of 10 mM AEBSF (ApexBio), and samples were then acidified with formic acid for analysis by mass spectrometry. For TATA, 100 μM synuclein in 50 mM sodium phosphate buffer was reacted with 0.5 mM TATA-^12^C_3_/^13^C_3_ (Creative Molecules). Samples were incubated for 5 minutes with 254 nm UV light from the same lamp as was used for the ABAS reactions. Samples were then split and digested with either proteinase K or trypsin at an enzyme: protein ratio of 1:20. For SDA reactions, 20 μL of 1mg/mL α-synuclein was crosslinked using 1 mM SDA (Creative Molecules, Inc.). Aliquots were incubated for 15 minutes in the dark prior to incubation under the same UV lamp as used previously for ABAS reactions but changing the wavelength to 366 nm. Samples were then run on an SDS-PAGE gel, and bands representing the α-synuclein monomer were excised and subjected to in-gel trypsin digestion. After in-gel digestion, samples were acidified using formic acid prior to mass spectrometric analysis. The CBDPS crosslinking reaction mixture consisted of 238 μL of 50 μM α-synuclein, with 0.12 mM CBDPS. Samples were split and digested with either proteinase K or trypsin at an enzyme: protein ratio of 1:10. Digests were quenched with 10 mM AEBSF and samples were enriched using monomeric avidin beads (Thermo Scientific). Enriched samples were acidified for mass spectrometric analysis using formic acid.

### LC-MS/MS analysis

Mass spectrometric analysis was then performed using a nano-HPLC system (Easy-nLC II, ThermoFisher Scientific), coupled to the ESI-source of an LTQ Orbitrap Velos or Fusion (ThermoFisher Scientific), using conditions described previously [11]. Briefly, samples were injected onto a 100 μm ID, 360 μm OD trap column packed with Magic C18AQ (Bruker-Michrom, Auburn, CA), 100 Å, 5 μm pore size (prepared in-house) and desalted by washing with Solvent A (2% acetonitrile:98% water, both 0.1% formic acid (FA)). Peptides were separated with a 60-min gradient (0–60 min: 4–40% solvent B (90% acetonitrile, 10% water, 0.1% FA), 60–62 min: 40–80% B, 62–70 min: 80% B), on a 75 μm ID, 360 μm OD analytical column packed with Magic C18AQ 100 Å, 5 μm pore size (prepared in-house), with IntegraFrit (New Objective Inc., Woburn, MA) and equilibrated with solvent A. MS data were acquired using a data-dependent method. The data dependent acquisition also utilized dynamic exclusion, with an exclusion window of 10 ppm and exclusion duration of 60 seconds. MS and MS/MS events used 60000- and 30000-resolution FTMS scans, respectively, with a scan range of m/z 400–2000 in the MS scan. For MS/MS, the CID collision energy was set to 35%. Data were analyzed using the ^14^N^15^N DXMSMS Match program from the ICC-CLASS software package [27]. SDA crosslinking data was analyzed using Kojak [28] and DXMSMS Match. For scoring and assignment of the MS/MS spectra, b- and y-ions were primarily used, with additional confirmation from CID-cleavage of the crosslinker where this was available.

### Differential surface modification

Chemical surface modification with pyridine carboxylic acid N-hydroxysuccinimide ester (PCAS) (Creative Molecules) was performed as described previously [29]. Briefly, α-synuclein was prepared at 50 μM in 8 M urea in PBS, pH 7.4 (unfolded state), or in only PBS (folded state). Either the light or the heavy form of the ^13^C-isotopically-coded reagent (PCAS-^12^C_6_ or PCAS-^13^C_6_) was then added to give a final concentration of 10 mM. Reaction mixtures were incubated for 30 minutes and quenched with 50 mM ammonium bicarbonate. Samples were then mixed at a 1:1 ratio, combining folded (PCAS-^12^C) with unfolded (PCAS-^13^C) samples, as well as in reverse as a control. Samples were acidified with 150 mM acetic acid and digested with pepsin at a 20:1 protein: enzyme ratio overnight at 37°C. After digestion samples were prepared for mass spectrometry analysis using C_18_ zip-tips (Millipore). Zip-tips were equilibrated with 30 μL 0.1% TFA, sample was introduced, then washed with 30 μL 0.1% TFA and eluted with 2 μL of 0.1% formic acid/50% acetonitrile. Samples were analyzed by LC-MS/MS as described above.

### Hydrogen/deuterium exchange

Top-down ECD-FTMS hydrogen/deuterium exchange was performed as described previously [30]. Briefly, protein solution and D_2_O from separate syringes were continuously mixed in a 1:4 ratio (80% D_2_O final) via a three-way tee which was connected to a 100 μm x 5 cm capillary, providing a labeling time of 2 s. The outflow from this capillary was mixed with a quenching solution containing 0.4% formic acid in 80% D_2_O from the third syringe via a second three-way tee and injected into a Bruker 12 T Apex-Qe hybrid Fourier Transform mass spectrometer, equipped with an Apollo II electrospray source. In-cell ECD fragmentation experiments were performed using a cathode filament current of 1.2 A and a grid potential of 12 V. Approximately 800 scans were accumulated over the m/z range 200–2000, corresponding to an acquisition time of approximately 20 minutes for each ECD spectrum. Deuteration levels of the amino acid residues were determined using the HDX Match program [31] (S1 Fig).

Synuclein UVPD spectra were collected on a Thermo Scientific Orbitrap Fusion Lumos Tribrid mass spectrometer equipped with a 2.5-kHz repetition rate (0.4 ms/pulse) 213 nm Nd:YAG (neodymium-doped yttrium aluminum garnet) laser (CryLas GmbH) with pulse energy of 1.5 ± 0.2 μJ/pulse and output power of 3.75 ± 0.5 mW for UVPD. The solution was exchanged with deuterium using the same three-way tee setup, although in this case a 50 μm x 7cm capillary provided a labeling time of ~1s. Spectra were acquired for 8 or 12 ms, and resultant spectra were averaged and used for the data analysis with the HDX Match program as above.

### Circular dichroism

CD spectra were recorded on Jasco J-715 spectrometer under a stream of nitrogen. The content of α-helical and β-sheet structures was calculated using BeStSel web server [32].

### Discrete molecular dynamics modelling

**Fig 2:**
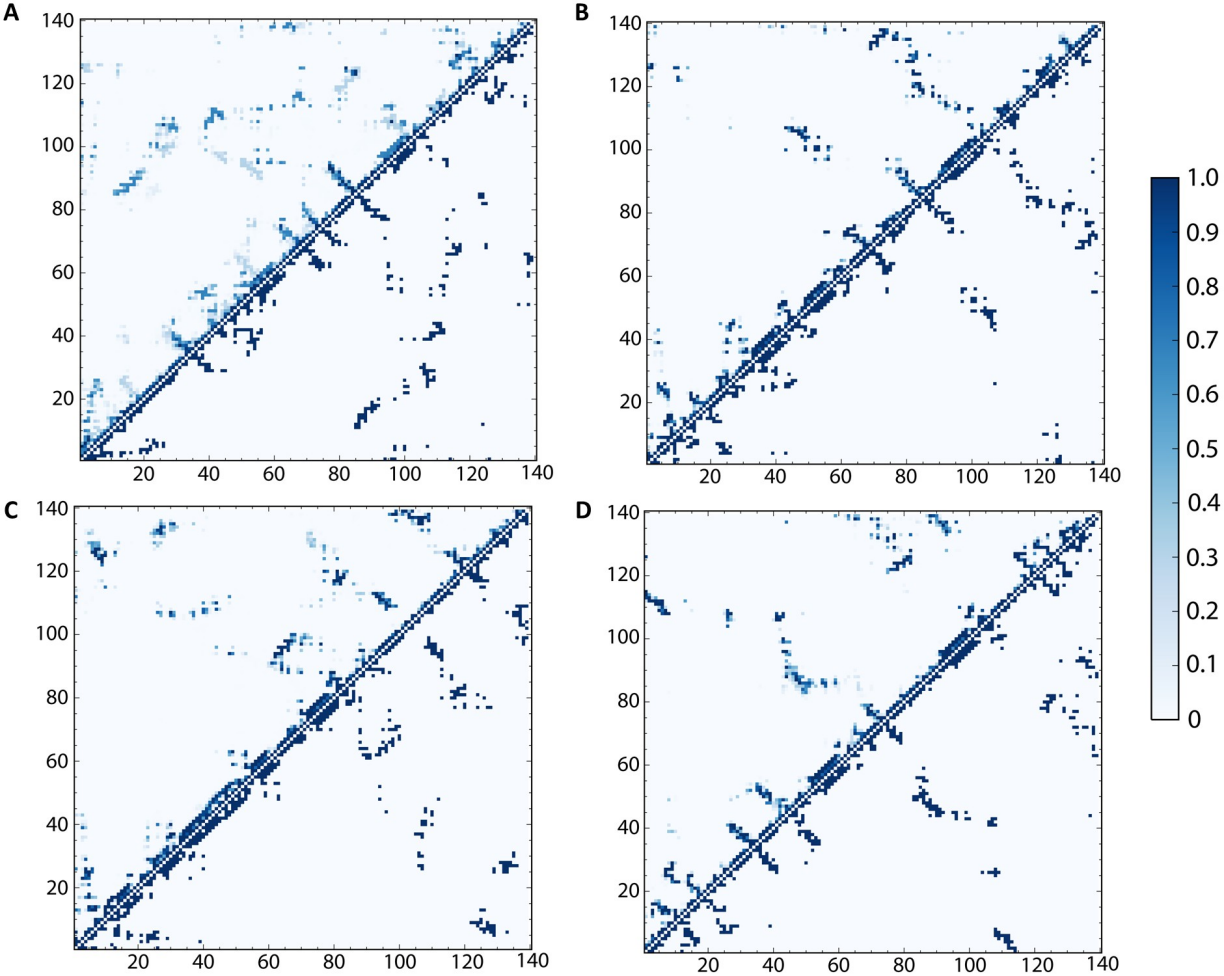
Contact frequency maps for representative clusters of α-synuclein model. Plots (A-D) represent static and frequency contact maps for the major representative clusters of protein structures during the simulations (populations: cluster 1 ~37%, cluster 2 ~28%, cluster 3 ~20%, cluster 4 ~8%). Points below the diagonals correspond to contacts between residues. Note that 0 means that the atoms are not in contact; 1 means that the two atoms are in contact. Two residues form a contact if their Cα atoms are within 8 Å of each other. Data points above the diagonal in plots (A-D) indicate how often each contact between two residues can be found within the clusters for which corresponding structure from bottom diagonals are centroids. The colour map quantifies how often these contact form, where white/zero means that contacts never form, and dark blue/one means that contacts always form).

**Fig 3:**
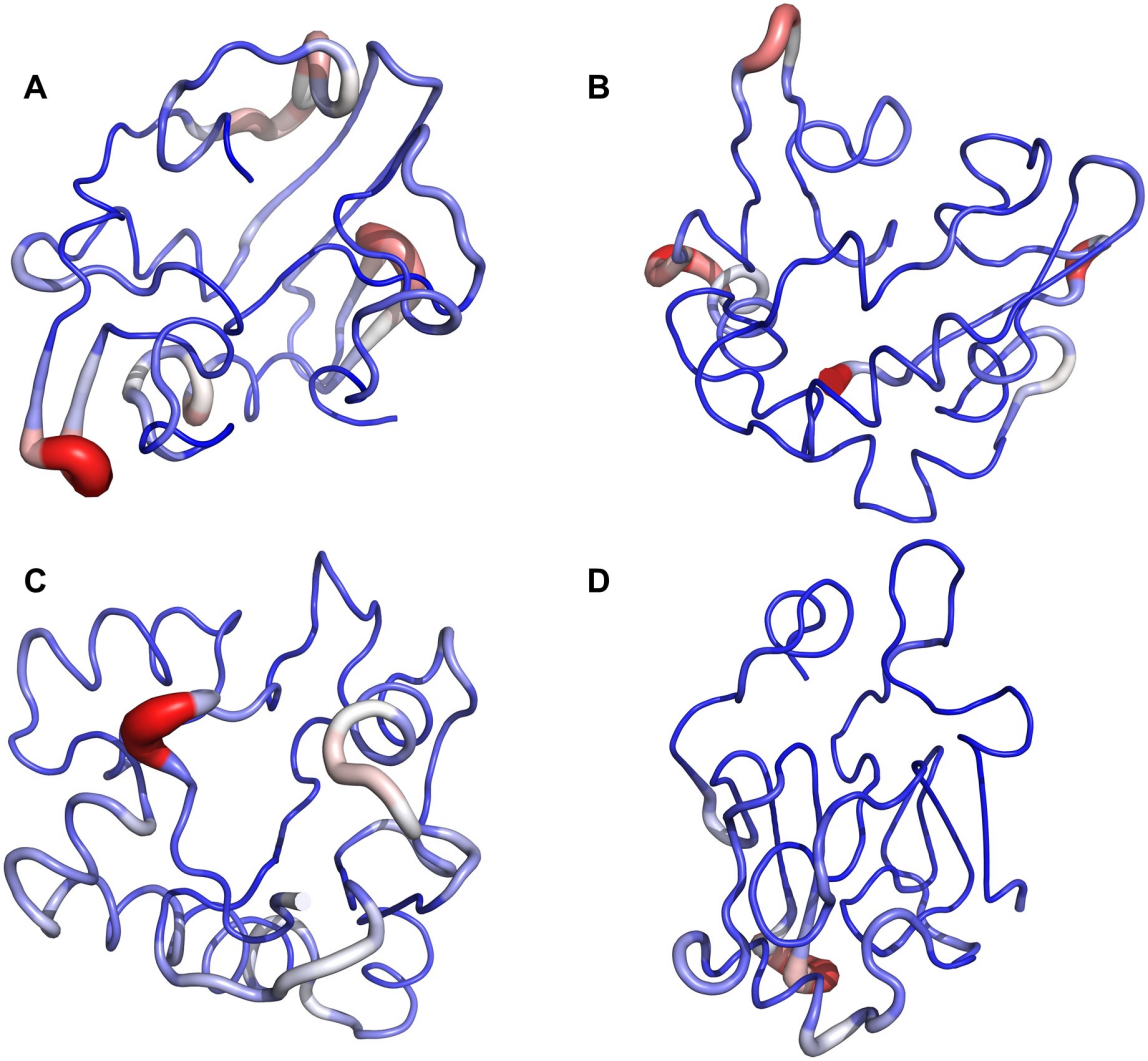
Tube representation of the fluctuations of the clusters. Tube models A-D represent the 37%, the 28%, the 20%, and the 8% clusters, respectively. The thickness and colour of the tubes indicate the dynamics of the corresponding regions within each cluster during the simulations. The tubes are coloured from blue (low flexibility) to red (high flexibility).

Crosslink guided discrete molecular dynamics (CL-DMD) simulations were performed according to the protocol described in our previous work [11]. Briefly, discrete molecular dynamics (DMD) is a physically based and computationally efficient approach for molecular dynamics simulations of biological systems [16, 17]. In DMD, continuous inter-atom interaction potentials are replaced with their discretized analogs, allowing the representation of interactions in the system as a series of collision events where atoms instantaneously exchange their momenta according to conservation laws. This approach significantly optimizes computations by replacing integration of the motion equations at fixed time steps with the solution of conservation-law equations at event-based time points [33]. In order to incorporate experimental data for inter-residue distances between corresponding atoms into DMD simulations, we introduced a series of well-shape potentials that energetically penalize atoms whose interatomic distance do not satisfy experimentally determined inter-atom proximity constraints. The widths of these potentials are determined by the cross-linker spacer length and side chain flexibility [11]. Starting from the completely unfolded structure of α-synuclein molecule, we performed an all-atom Replica Exchange (REX) [34] simulations of the protein where 24 replicas with temperatures equally distributed in the range from 0.375 to 0.605 kcal/(mol k_B_), are run for 6 x 10^6^ DMD time steps (S2 Fig). The simulation temperature of each of the replicates periodically exchanged according to the Metropolis algorithm allowing the protein to overcome local energetic barriers and increase conformational sampling. During the simulations we monitored the convergence of the system energy distribution specific heat curve, calculated by Weighted Histogram Analysis Method (WHAM) [35] which was used as the indicator of system equilibration. We discarded the first 2 x 10^6-time^ steps of system equilibration during the analysis. Next, we ranked all of the structures among all of the trajectories, and selected the ones with lowest 10% of the energies, as determined by the DMD Medusa force field [36]. These structures were then clustered using the GROMACS [37] distance- based algorithm described by Daura et al. [38]. It uses root-mean-square deviation (RMSD) between backbone Cα atoms as a measure of structural similarities between the cluster representatives. A RMSD cut-off was chosen to correspond to the peak of the distribution of pair-wise RMSDs for all of the low-energy structures. Because the energies of the resulting centroids representative of the clusters are very close to each other (S3 Fig) and picking one of them would potentially introduce a bias related to our scoring energy function, we presented them all as our predicted models of the α-synuclein globular structure. We then calculated the root-mean-square deviation of atomic positions within each cluster and used this as a measure of fluctuations of the structures of corresponding centroids (Figs 2 and 3). In order to obtain information on the global folding of α-synuclein, we performed clustering analysis on the lowest-energy structures obtained during CL-DMD simulations.

## Results and discussion

**Fig 4:**
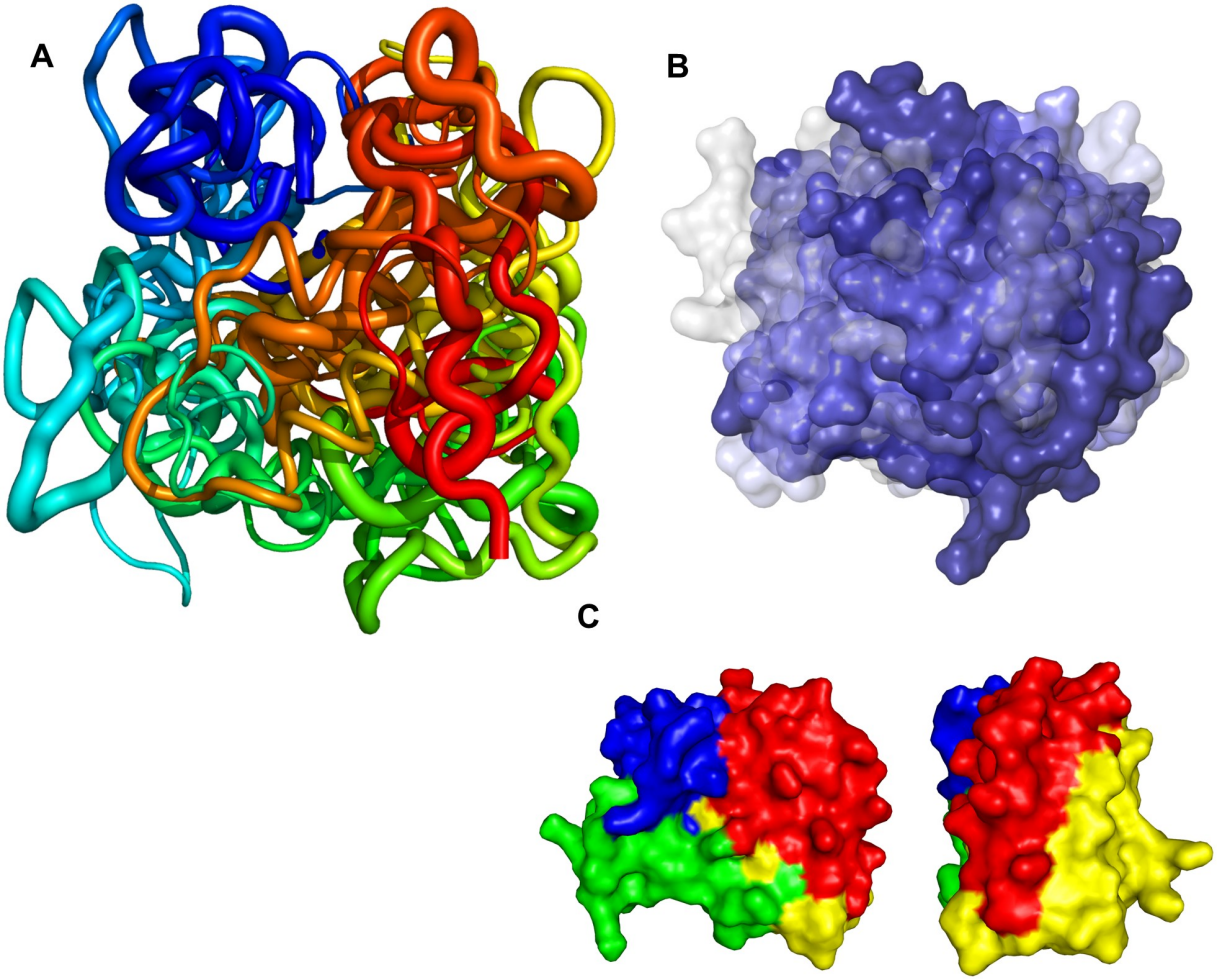
Structure of native α-synuclein in solution as determined by CL-DMD. **A.** Conformational ensemble of α-synuclein. Representatives of the four major clusters are aligned and coloured N-to-C from blue-to-red. The thickness of the cartoon representations corresponds to the content of approximately 37%, 28%, 20% and 8% of the overall population of the structures in each cluster. **B.** Surface representation of the ensemble. Representatives of the four major clusters as in panel A are aligned and coloured blue-to-white according to percent of all structures in each cluster. **C.** Subdomain topology of the α-synuclein conformational ensemble. Residues are coloured 1–27 blue, 28–52 green, 53–108 yellow, 109–140 red.

In contrast to ordered proteins, where the lowest energy structures are usually represented by a few centroids with distinct conformations and folds close to the corresponding native structure [11], disordered proteins are usually represented by a broader variety of structures, which are distinct from each other, reflecting the conformational freedom of the intrinsically disordered protein [39]. In our case, the lowest energy structures forming the conformational ensemble for α-synuclein are shown as overlays in Fig 4A. They can be described as rather compact globular structures with a common general topology, containing a few distinct secondary structure fragments. Overall, a distinct topology can be observed for all these conformers (Fig 4B). The protein forms a three-prong closed claw-like shape with the N-terminal (blue), intermediate (yellow), and C-terminal (red) subdomains having converged at the top, with the connecting subdomain (green) being located at the bottom of the structure (Fig 4). The C-terminal portion of the molecule was found in close proximity to the N-terminal portion, possibly reflecting long-range electrostatic interactions between these subdomains (Fig 4) [8]. The C-terminal portion was also in contact with the intermediate subdomain—in some conformers it protruded deeper into the structure and was positioned between the N-terminal and the intermediate subdomains. It should be noted that non-N-acetylated α-synuclein protein was used in this study, although in the cell, α-synuclein is predominantly N-acetylated [40], and this post-translational modification may have some effect on the protein structure by increasing the propensity of the N-terminus to form an α-helix [41], and that this may affect the aggregation of synuclein under some conditions [42, 43]. N-terminal acetylation, however, does not appear to have a significant effect on the propensity of synuclein to aggregate [44] under lipid-free conditions, and it therefore seems that the structural features important for predicting aggregation and designing small molecules to affect misfolding are also present in the absence of acetylation.

Some of the experimental constraints may conflict with each other due to the fact that proteins exist in multiple conformations. During DMD simulations, the total potential is minimized, satisfying an optimal number of constraints and generating an ensemble of structures satisfying subsets of consistent experimental constraints. This ensemble is further clustered into substates that represent subsets of non-conflicting constraints. Depending on the conformation and the number of constraints that are satisfied, these structures will have different energy scores assigned by the force field [16, 36]. This allows us to account for both the nature of conformational changes of the protein in solution (i.e., while the structure of the protein is “breathing” and transitioning between conformations), and possible errors during the determination of the experimental constraints [12]. Nevertheless, we found that the lowest-energy conformers satisfy most of the experimental distance constraints (S4 Fig), while the models with higher energy satisfy a lower number of constraints. No quantitative crosslinking data or weighting of observed crosslinks were used in the simulations, but more-populated conformations have a higher chance of being represented in the resulting crosslinking dataset, given that crosslinks are not randomly distributed and are most likely to form between residues which spend a significant amount of time within close contact. A comparison of predicted structures within the ensemble revealed that the conformational changes to the protein structure were mainly due to movement of the large loops and hairpins constituting the N-, C-terminal, and intermediate subdomains.

The validity of this ensemble was tested by removing all of the crosslinking constraints and allowing the structure to relax over 2x10^6^ steps, to see if the structure was stabilized only by the potentials enforcing satisfaction of the experimental crosslinks. The predicted representatives of the clusters were allowed to fluctuate in this way, and the result was a relatively minor expansion of the protein (results for the centroid of the most representative lowest energy cluster, Fig 3A, are shown for clarity), from a radius of gyration of 14.1 Å initially, to an average of 15 Å (S5 Fig). This indicates that the structure has not been overfit to the experimental data during the simulation, which can be a problem when modelling intrinsically disordered proteins with experimental distance constraints [14].

The crosslinking analysis applied here is conceptually similar to the PRE-NMR [8, 40, 45, 46] or FRET [47, 48] techniques used for the prediction of α-synuclein conformational ensembles, with pairwise distances between crosslinked residues being analogous to the pairwise distances between the spin-label and the atomic nuclei of the protein in case of PRE-NMR (S6 Fig) or between fluorophores, in the case of FRET. The lowest energy-cluster centroids in our ensemble tend to be more compact than those based on PRE-NMR (S6 and S7 Figs), FRET ensembles, or SAXS data. It has been suggested that the higher values of the radius of gyration determined by these techniques may be caused by an existing equilibrium between the monomeric and multimeric states of the synuclein protein under the experimental conditions used [49, 50]. SAXS data can be uniquely sensitive to the more-extended conformations of the ensemble [49]. Unconstrained all-atom molecular dynamics simulations produced multimodal distributions which included compact states [49], although it was noted that modelling disordered proteins may require the development of specialized force fields [43, 49]. It was also shown that the addition of experimental distance constraints to the all-atom Monte Carlo conformational search leads to more compact structures [49]. Thus, representation of the slightly more compact states may still be caused by bias due to the short-range crosslinking reagents used in this study, as well as by the traditional parameterization of the Medusa force field [15–17].

In addition, we selected the lowest-energy structures for our clustering and model selection, which eliminated some of the unfolded less-compact states, while in the PRE-NMR based approach, the authors used all of the generated structures for the analysis. We are confident in this approach as these lower energy conformations were also compatible with the long-distance crosslinks. The other difference between these two techniques is the range of distance constraints produced. The PRE-NMR technique uses distances up to ~2 nm. Local residue-specific conformational preferences are not well reproduced in the PRE-RMD ensembles [46]. When applying CL-DMD for well-structured model proteins, i.e., proteins with known crystal structure, we also found that long-distance constraints (>15 A) were not particularly helpful in finding the true protein structure solutions [11]. Short-distance constraints were critical in finding the correct local conformations in well-structured proteins and might possibly be helpful in producing more detailed structures in the case of the CL-DMD ensembles described here.

**Fig 5:**
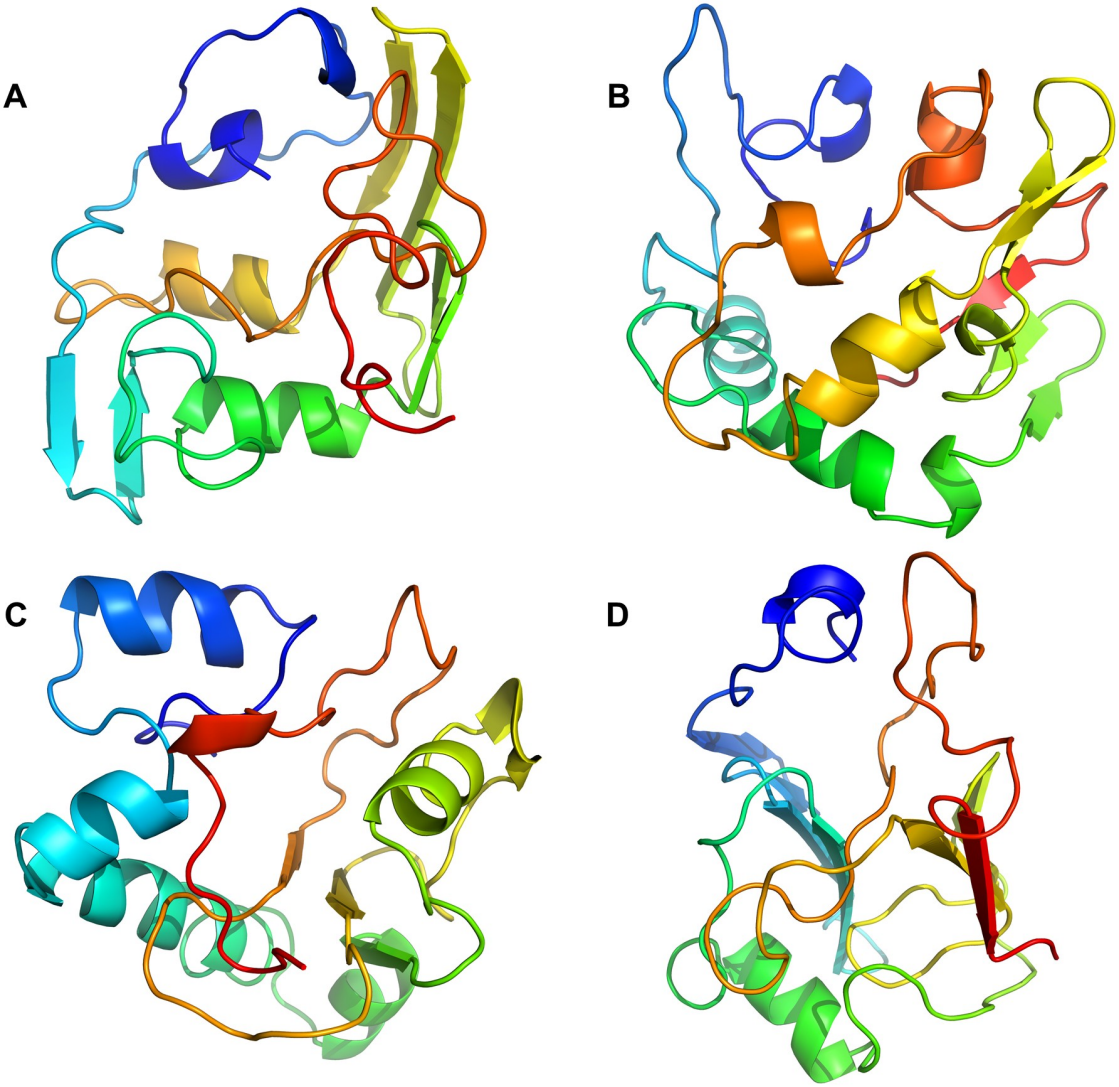
Comparison of the transient secondary structure in the α-synuclein conformational ensemble. Shown here are representative conformers of the α-synuclein ensemble shown in Fig 4. Structures A, B, C, and D represent clusters containing approximately 37%, 28%, 20%, and 8% of the overall population of the structures, respectively. Structures are coloured from blue to red, from the N- to C-terminus; the NAC region 61–95 corresponds to the light green-to-yellow section.

Some transient secondary-structure elements were observed in the conformers of the ensemble (Fig 5). The extent and the location of the predicted secondary-structure motifs in the lowest-energy conformer were in good agreement with the experimental data obtained by HDX and CD. Thus, ~25 of the total number of protected protons were detected by HDX (Fig 1) of the whole protein, and ~2.4% of α- and 29.1% of β-structure was detected by CD. We were able to detect amide HDX protection only when the exchange time was reduced from ten seconds to two seconds, which indicates the transient nature of the secondary structure observed. The location of the predicted secondary structure within the N-terminal portion of the protein was confirmed by determining the protection status of individual residues using the c-ion fragment series (i.e., the fragments starting from N-terminus) as obtained by ECD MS/MS (Fig 1 and S1 Fig). Here, we observed some propensity for the protein to form α-helical secondary structure near the N-terminus, particularly between residues 25–55. However, it is not nearly as extensive as the secondary structure formed in the presence of detergent micelles. In particular, two centroids (Fig 5B and 5C) show a greater tendency towards α-helicity, and together represent some 48% of the ensemble.

Unfortunately, for this protein, using ECD or ETD fragmentation we were unable to obtain reliable z-ion fragment series (i.e., fragments starting from the C-terminus) which would have spanned the predicted β-structural elements, probably due to the high net negative charge on the C-terminal portion of the molecule. However, the amount of protection predicted in the structure of the C-terminal part of the model, added to observed protection of the N-terminal part from ECD-HDX data, is in agreement with the total protection value of the protein (Figs 1 and 3, S8 Fig).

**Fig 6:**
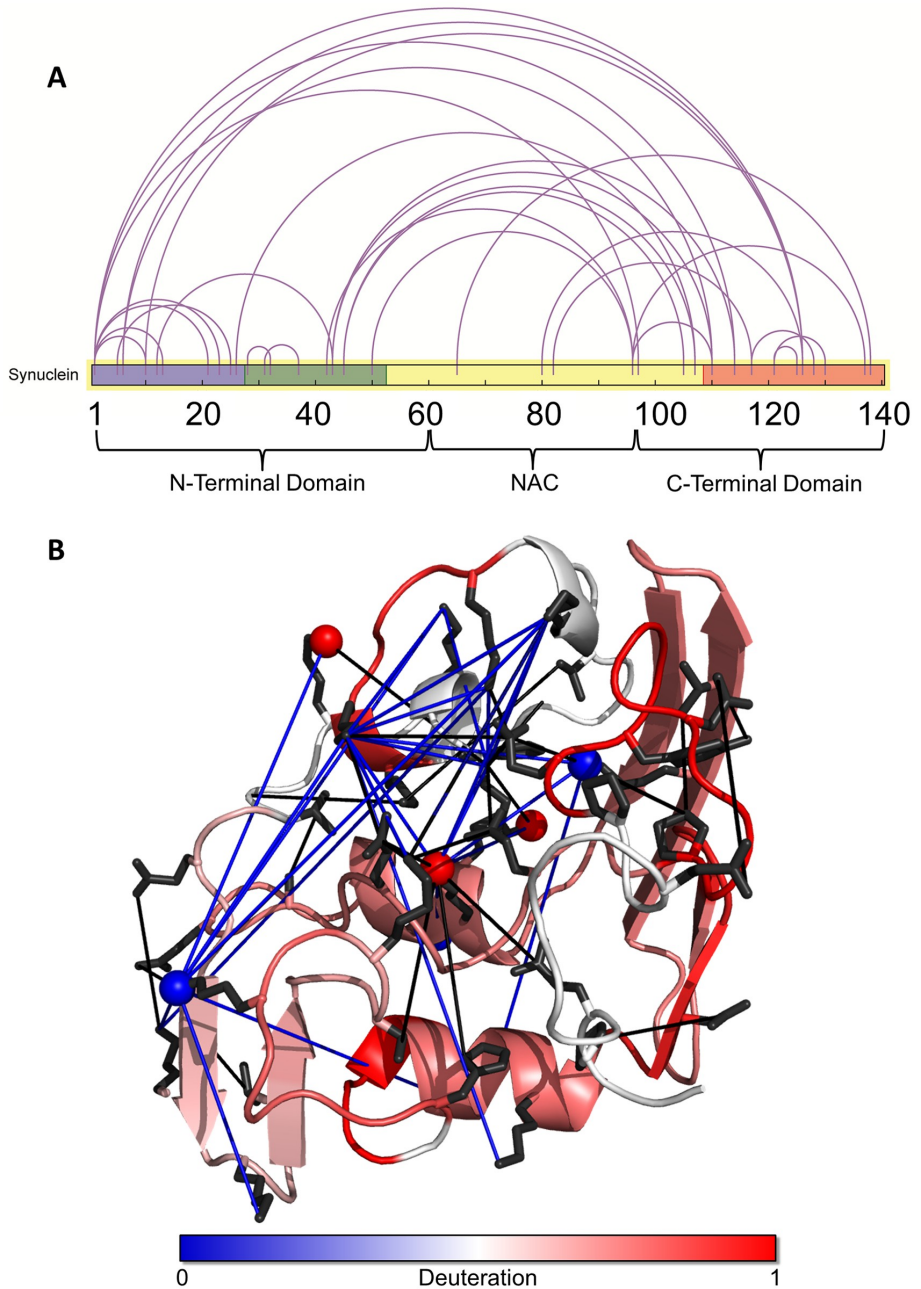
Experimental validation of the α-synuclein structure with SM, HDX, and LD-CL. **A.** A schematic diagram of the synuclein protein which each crosslink used for the CL-DMD simulation shown as a line. The diagram is coloured in accordance with the subdomain topology described in Fig 4. **B.** The lowest energy conformer from the ensemble as in Fig 1 is shown, with crosslinks and other validating data indicated on the structure. Short-distance crosslinks used in the CL-DMD simulations are shown as black lines. The main chain is coloured from blue to red according to the experimentally observed backbone amide protection values from the HDX experiments. Atoms that are equally modified with PCAS in the native and denatured states are shown in blue; atoms that are preferentially modified after denaturation with 8M urea atoms are shown in red. LD-CL CBDPS crosslinks are shown as blue lines.

We then used our recently developed top-down UVPD-HDX method [51] in an attempt to determine the possible locations of secondary structure in the C-terminal portion of the α-synuclein molecule. We were able to observe a number of UVPD-specific fragment ions spanning the C-terminal portion of the protein sequence—a region which had previously not been covered. Deuteration analysis of these fragments allowed us to confirm the presence of secondary structure in this region and to further narrow the location of the secondary-structure elements to residues 116–119 and 128–138, and ~10 protected residues in the residue 71–116 segment of the sequence (Figs 1 and 3, S1 and S8 Figs). Surface modification results were in agreement with the final α-synuclein structures. As expected, lysine residues which ended up being exposed to the solvent in the model were equally modified with PCAS in the unfolded and folded states (i.e., with and without 8 M urea), and residues which were partially buried in the model exhibited a larger extent of modification in the fully unfolded state with 8 M urea (Fig 6). The long-distance intra-protein CBDPS crosslinks were also in good agreement with the final models of the protein.

Interestingly, the aggregation-prone non-amyloid-β component (NAC) region, which is reportedly involved in the nucleation of the aggregation [52], was consistently predicted to contain hairpins in extended conformation, which were in contact with the C-terminal loop, which, in turn, interacted with the N-terminal sub-domain. It would be intriguing to see if a distortion of any of these interactions could lead to an “opening up” of this region which might facilitate stacking interactions with similar portions of other monomers during the aggregation initiation. We are currently performing CL-DMD-based differential structural characterization of α-synuclein oligomers, in comparison to the native structures reported here, in order to localize critical mis-folding events leading to the pathological aggregation. Two recent studies, by Lautenschlager *et al*. [4] and Fusco et al [5] have indicated that cellular toxicity of synuclein oligomers may be dependant on both the N- and C-terminal domains and their interactions with lipid bilayers. In toxic oligomeric species, the synuclein N-terminus has less interaction with the membrane surface, and this interaction is replaced by an interaction with the NAC region, leading to toxicity. In the case of the C-terminus, increased concentrations of calcium may create a charge-neutralizing effect, which leads to increased toxicity and oligomer formation, possibly by disrupting its interaction with the NAC region.

The β-structure in the NAC-containing intermediate domain and in the C-terminal subdomain of the predicted ensemble is in general agreement with the C-terminal half of the protein’s propensity towards formation of β-structure, as determined by NMR [7]. The fact that, in the ensemble, the NAC region was repeatedly found to contain β-structure-like hairpins may explain the propensity of this region to form inter-molecular β-structures which appear early in the aggregation process. These hairpins also resemble those observed in the aggregating form of Aβ1–40 found in fibrils [53], and in the mature fibrillar form of the synuclein protein [54]. This increases our confidence in the significance of this particular transient structure in the native protein. In agreement with previous studies [8, 45], we also found that the NAC region was in contact with C- terminal portion of the molecule, but in our ensemble it is not quite shielded by it, as was suggested. Rather, it is stabilized by β-sheet-like contacts. Additionally, the residues V74-V82 within the NAC region, which has been shown to be critical for incorporation into newly forming fibrils (Ricardo *et al*., 2018), remains somewhat exposed in the most favorable conformations of the ensemble, and with this region already incorporating important β-sheet contacts, it seems primed for incorporation into newly-forming fibrils, which may help explain the tendency for native synuclein to spontaneously form fibrils with mere agitation.

The structures of the major α-synuclein conformational clusters determined here and the presence of the secondary structure detected in the NAC region and C-terminal portions of the molecule allowed us to hypothesize a possible mechanism for the misfolding conformational change and the early aggregation events. The existence of the transient hairpin/beta-structure in the NAC and C-terminal subdomains predisposes the protein to form the beta-nucleation sites of the early oligomers, which may further mature to the cross-beta-structure of the fibrils. The mis-folding conformational change that is necessary for the formation of inter-molecular contacts would then be the detachment of the NAC-containing hairpin (possibly in concert with stabilizing C-terminal beta-strands) from the core of the molecule. This would provide a stacking template for the similarly detached NAC hairpin of the other α-synuclein molecule. This hypothesis can be tested by determining the structure of the early oligomers, which is currently underway in our laboratories.

**Fig 7:**
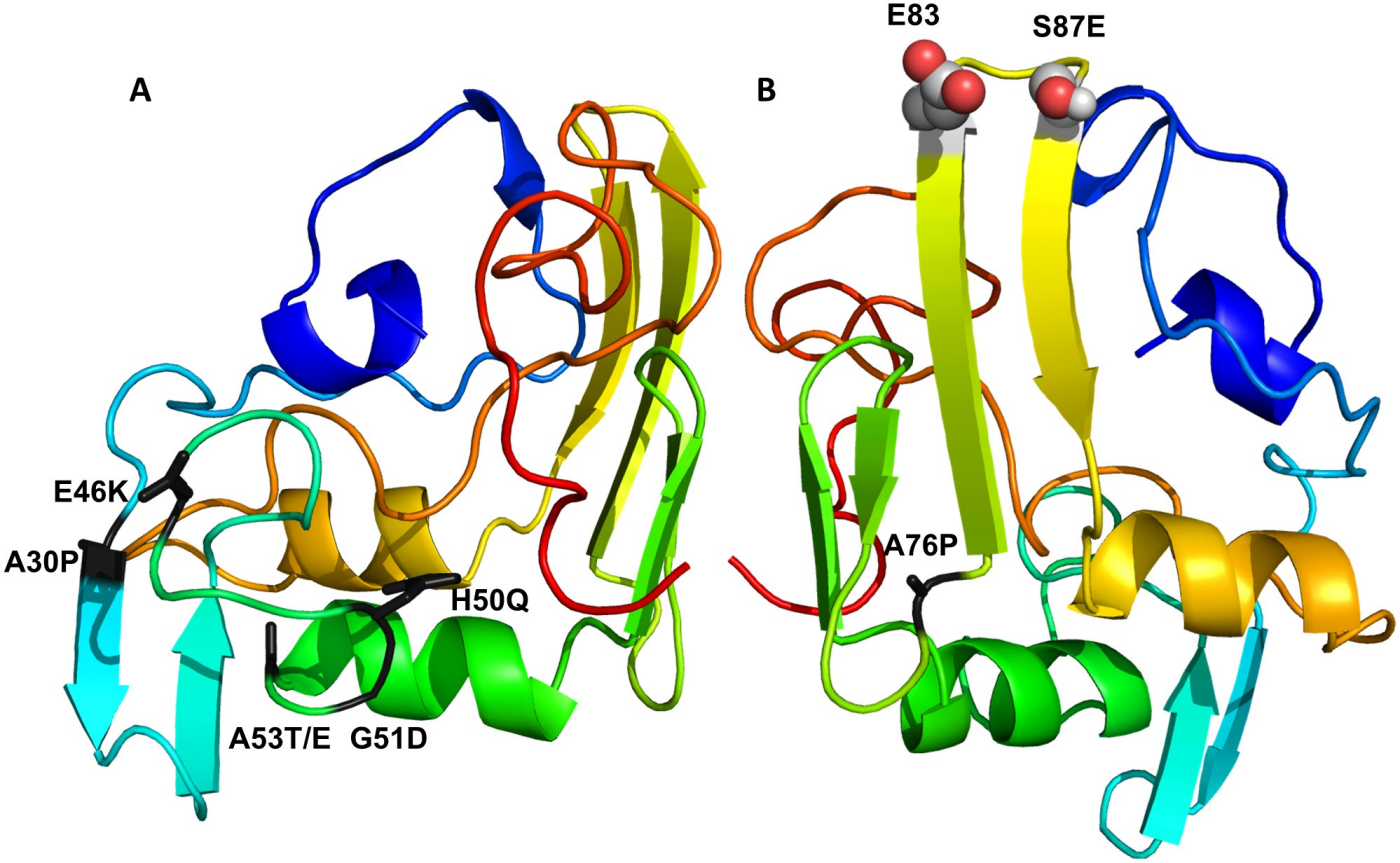
Location of the mutations influencing aggregation of α-synuclein. A. Familial mutations located in the connecting subdomain are highlighted in black in the centroid structure of the cluster 1. B. Mutations potentially influencing the NAC β-hairpin.

The locations of the mutations shown to be influencing the aggregation of the α-synuclein within the proposed structure is interesting (Fig 7) [55]. The familial mutations A30P, E46K, H50Q, G51D and A53 T/E [56] were found to located on the same surface of the connecting subdomain. Although the effects of the these mutations could possibly be produced via different mechanisms (such as modified membrane binding or fibril stability), at least the effect of the oligomer-promoting mutations A30P and A56P can be explained in terms of the structure described here, where they would increase the segmental flexibility and possibly relax the location and interaction of the N-terminal subdomain with the core of the molecule. A76P oligomer-promoting mutation similarly would facilitate segmental flexibility and the detachment of the NAC-containing β-hairpin postulated above. The S87E mutation, which blocks α-synuclein oligomerization and fibrilogenesis [57], would possibly disrupt NAC β-hairpin via electrostatic repulsion with the E83 residue.

### Conclusions

In summary, we have determined *de novo* the conformational ensemble of native α-synuclein in solution by short-distance crosslinking constraint-guided DMD simulations, and validated this structure with experimental data from CD, HDX, SM, and LD-CL experiments. The predicted conformational ensemble is represented by rather compact globular conformations with transient secondary structure elements. The obtained structure can serve as a starting point for understanding the mis-folding and oligomerization of α-synuclein.

## Supporting information

S1 Fig Hydrogen-deuterium exchange of α-synuclein. **A.** The number of protected hydrogen atoms in c-ions detected by ECD-FTICR mass spectrometry. The upper blue line represents the theoretical situation of perfect protection of all backbone hydrogen atoms. The lower line represents the number of actual protected hydrogen atoms detected at that fragment. **B.** The deuteration status of backbone amides from ECD data. A value of 1 indicates that the residue is unprotected; a value of zero represents complete protection from exchange with deuterium. **C.** The number of protected hydrogen atoms in fragment ions detected by UVPD-FT mass spectrometry. **D.** The deuteration status of backbone amides from UVPD data. N-terminal and C-terminal fragment data are shown in blue and red, respectively.
(TIF)

S2 Fig Replica exchange (REX) rate illustration. The plot shows the exchange rates between 24 replicas during DMD REX simulation. Each replicate is represented by line of its own colour (from red to blue). Each 24 of the replicates starts with temperatures equally distributed in the range from 0.375 to 0.605 kcal/(mol k_B_) and is run for 6 x 10^6^ DMD time steps. The first 2 x 10^6^ DMD time steps were disregarded during the analysis to account for equilibration of the simulation. During the simulation, replicates exchange their temperatures allowing “colder” replicates to “heat up” and “hot” replicates to “cool down” (lines moving up and down in the plot), which assists in overcoming small local energy barriers. As it can be seen, each replicate (line of each colour) passes through different temperatures from low to high, which indicates that all replicates are well mixed (the plot is highly mosaic) during the simulation. For each replicate, states with different temperatures are visited during REX simulation, which suggests that the REX analysis is adequate.
(TIF)

S3 Fig Heat capacity curve of native α-synuclein. The heat capacity (Cv) curve was computed using WHAM [58] on the REX/DMD trajectories for α-synuclein in the range of 0.375 to 0.605 kcal/(mol kB) DMD temperature units. The large peak corresponds to the unfolding temperature of the protein. States corresponding to all representative structures are located on the wide shoulder of the curve on the left. The absence of major peaks in this area indicates that the protein can coexist in multiple compact states.
(TIF)

S4 Fig Comparison of crosslinking constraints satisfied by each cluster. The distances at which a crosslink is considered satisfied for ABAS, EDC, SDA, and TATA crosslinks are < 17 Å, < 10 Å, < 15 Å, and < 14 Å, respectively. Unsatisfied crosslinks were counted in the simulation as a penalty.
(TIF)

S5 Fig Synuclein structure fluctuations in the absence of crosslinking constraints. The centroid structure from the lowest energy cluster in the ensemble (Fig 3A) was allowed to relax during simulation without any crosslinking constraints at the temperature of 0.45 (kcal/mol/kb DMD units) for 2 x 10^6^ DMD time steps. The first 500k steps were discarded as the system equilibration.
(TIF)

S6 Fig Comparison of the CL-DMD and PRE-NMR ensembles. PRE-NMR α-synuclein ensemble corresponding to the publication by Allison et al., JACS 2009 [59] was obtained from Protein Ensemble Database http://pedb.vib.be. Ensemble average inter-residue distances from the residues, which were spin labeled in the PRE-NMR study. Each graph shows the average inter-residue differences between all residues in the ensemble and the spin-labelled residues in the PRE-NMR experiment. Panels **A-D** correspond to the four major CL-DMD clusters presented in this work, the **E** panels correspond to the PRE-NMR ensemble.
(TIF)

S7 Fig Comparison of the CL-DMD and PRE-NMR ensembles’ radii of gyration probabilities. Radius of gyration (Rg) probability distributions for the ensemble of α-synuclein structures obtained by CL-DMD (combination of four major clusters) and for PRE-NMR structures downloaded from Protein Ensemble Database http://pedb.vib.be.
(TIF)

S8 Fig Comparison of top-down HDX data from UVPD fragmentation of hydrogen/deuterium exchanged synuclein with the expected protection for each cluster. The expected protection is represented by blue boxes. The deuteration levels determined from the HDX experiments are shown using a blue-white-red gradient, with blue at 0 (protected from exchange) and red at 1 (exchanged).
(TIF)

S1 Table Crosslinks used for molecular modelling. Crosslinks found which were used during CL-DMD simulations. *These crosslinks were previously reported in [25].
(XLSX)

S2 Table Surface modification of α-synuclein. Residues modified by PCAS-^12^C_6_^13^C_6_ in a urea-PCAS surface modification experiment. H/L ratios measure the surface exposure of a residue by comparing the ratio of native to urea-treated proteins modified by PCAS-^12^C_6_^13^C_6_. A ratio of over 1.5 indicates that the residue in question is somewhat protected in the native structure.
(XLSX)

S3 Table Long distance CBDPS crosslinking of α-synuclein. Long-distance crosslinks detected using CBDPS crosslinker. All crosslinks were within the 25Å distance maximum for CBDPS.
(XLSX)
