# Supplementary figures and images for: Conformational ensemble of native α-synuclein in solution as determined by short-distance crosslinking constraint-guided discrete molecular dynamics simulations
Source: PLoS Comput Biol. 2019 Mar 27;15(3):e1006859. doi: 10.1371/journal.pcbi.1006859 (PMC6453469; doi:10.1371/journal.pcbi.1006859)

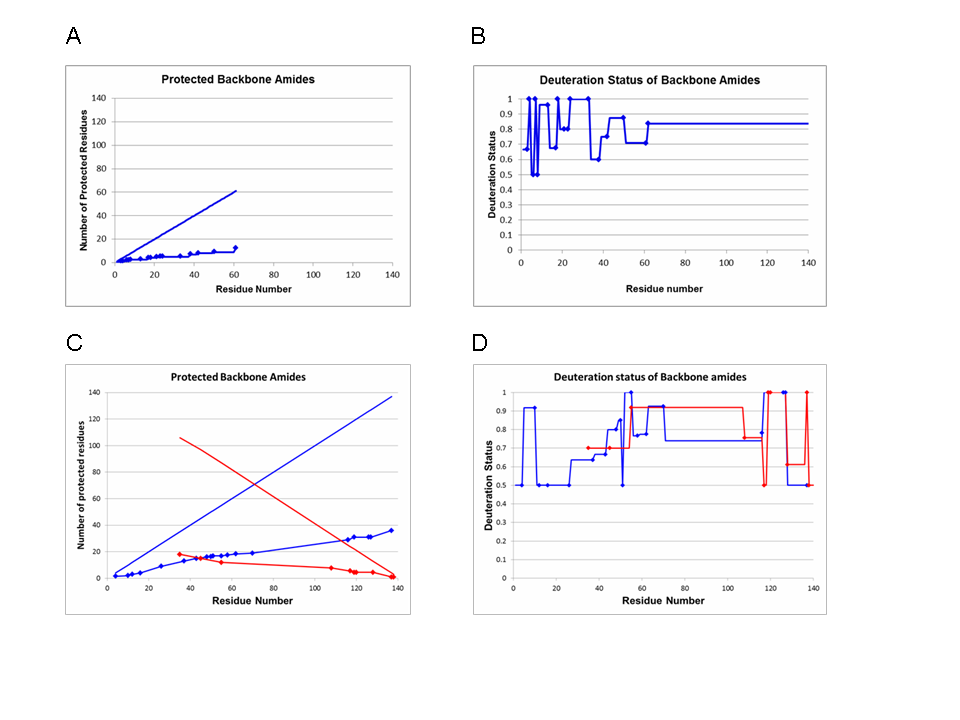

Supplement: S1 Fig — A. The number of protected hydrogen atoms in c-ions detected by ECD-FTICR mass spectrometry. The upper blue line represents the theoretical situation of perfect protection of all backbone hydrogen atoms. The lower line represents the number of actual protected hydrogen atoms detected at that fragment. B. The deuteration status of backbone amides from ECD data. A value of 1 indicates that the residue is unprotected; a value of zero represents complete protection from exchange with deuterium. C. The number of protected hydrogen atoms in fragment ions detected by UVPD-FT mass spectrometry. D. The deuteration status of backbone amides from UVPD data. N-terminal and C-terminal fragment data are shown in blue and red, respectively. (TIF) [file pcbi.1006859.s001.TIF]

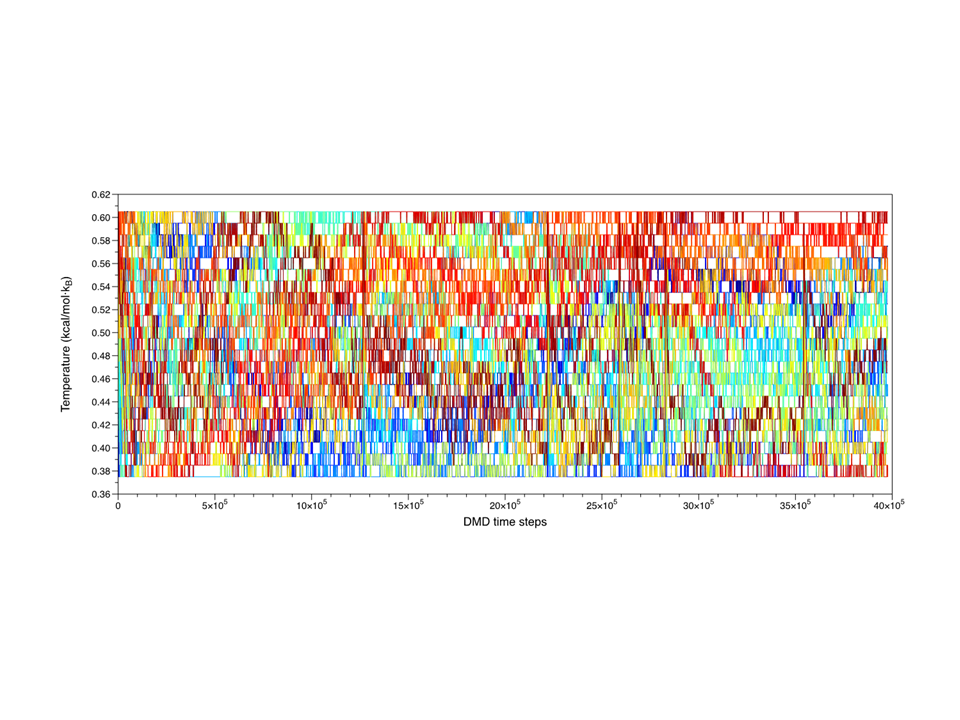

Supplement: S2 Fig — The plot shows the exchange rates between 24 replicas during DMD REX simulation. Each replicate is represented by line of its own colour (from red to blue). Each 24 of the replicates starts with temperatures equally distributed in the range from 0.375 to 0.605 kcal/(mol kB) and is run for 6 x 10^6^ DMD time steps. The first 2 x 10^6^ DMD time steps were disregarded during the analysis to account for equilibration of the simulation. During the simulation, replicates exchange their temperatures allowing “colder” replicates to “heat up” and “hot” replicates to “cool down” (lines moving up and down in the plot), which assists in overcoming small local energy barriers. As it can be seen, each replicate (line of each colour) passes through different temperatures from low to high, which indicates that all replicates are well mixed (the plot is highly mosaic) during the simulation. For each replicate, states with different temperatures are visited during REX simulation, which suggests that the REX analysis is adequate. (TIF) [file pcbi.1006859.s002.TIF]

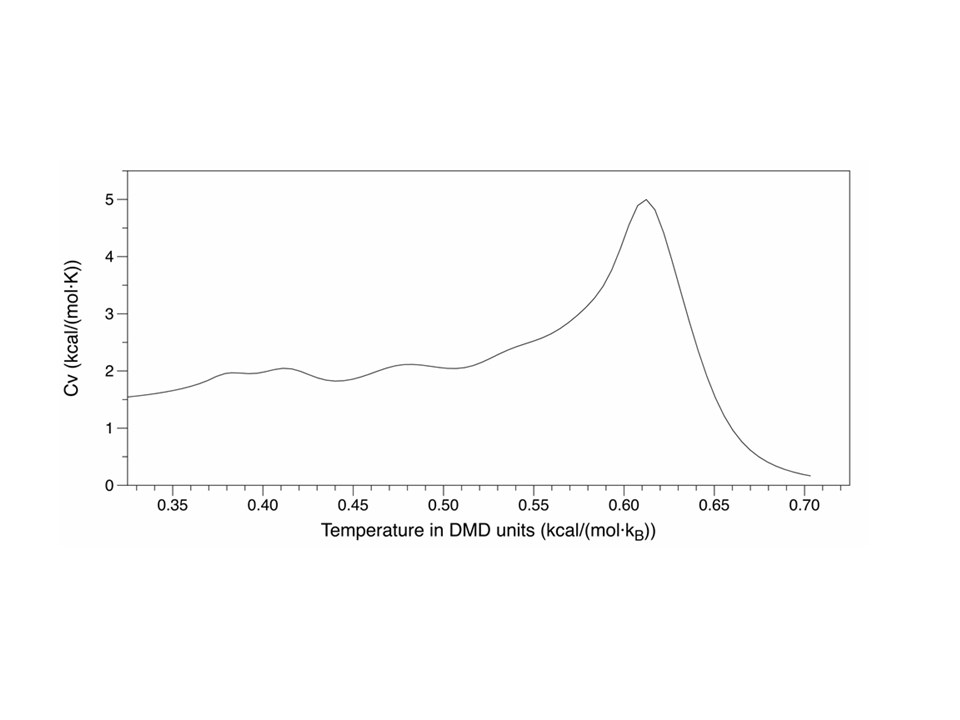

Supplement: S3 Fig — The heat capacity (Cv) curve was computed using WHAM [58] on the REX/DMD trajectories for α-synuclein in the range of 0.375 to 0.605 kcal/(mol kB) DMD temperature units. The large peak corresponds to the unfolding temperature of the protein. States corresponding to all representative structures are located on the wide shoulder of the curve on the left. The absence of major peaks in this area indicates that the protein can coexist in multiple compact states. (TIF) [file pcbi.1006859.s003.TIF]

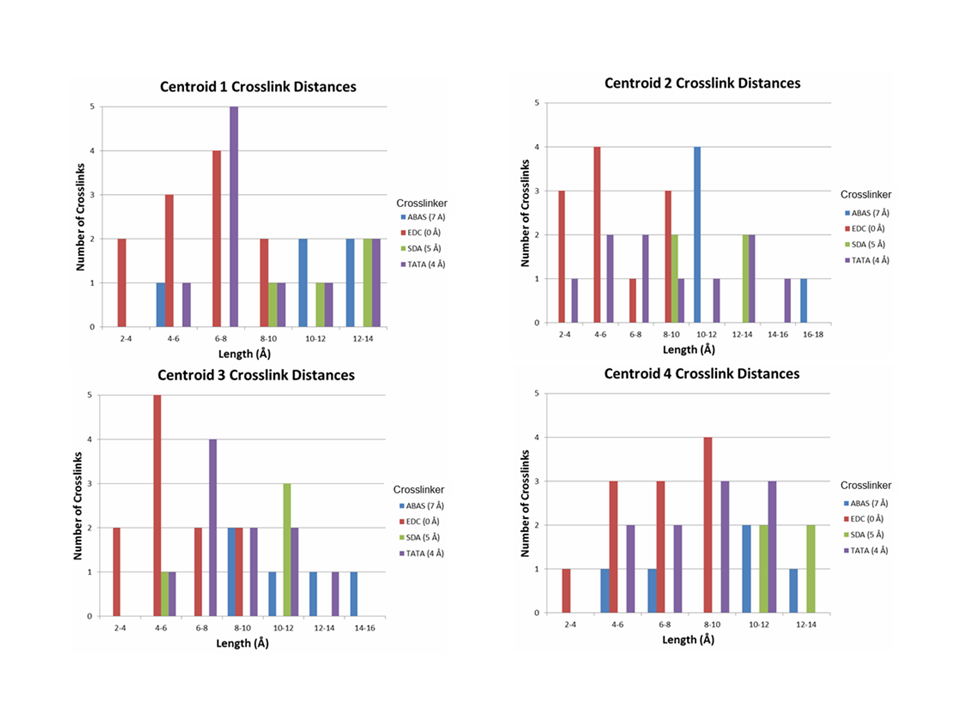

Supplement: S4 Fig — The distances at which a crosslink is considered satisfied for ABAS, EDC, SDA, and TATA crosslinks are < 17 Å, < 10 Å, < 15 Å, and < 14 Å, respectively. Unsatisfied crosslinks were counted in the simulation as a penalty. (TIF) [file pcbi.1006859.s004.TIF]

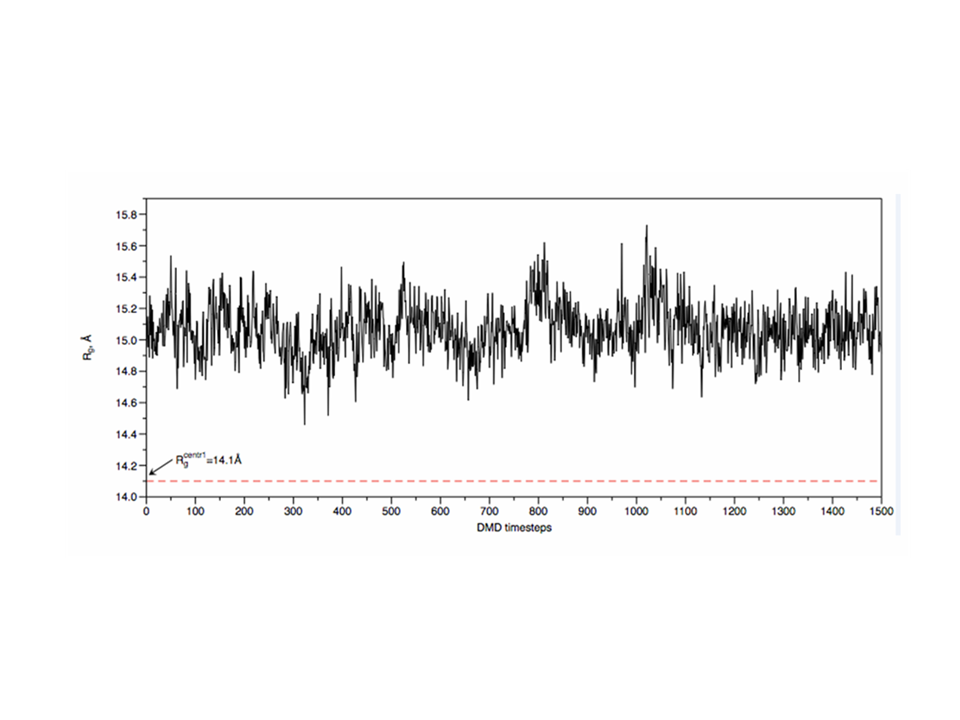

Supplement: S5 Fig — The centroid structure from the lowest energy cluster in the ensemble (Fig 3A) was allowed to relax during simulation without any crosslinking constraints at the temperature of 0.45 (kcal/mol/kb DMD units) for 2 x 10^6^ DMD time steps. The first 500k steps were discarded as the system equilibration. (TIF) [file pcbi.1006859.s005.TIF]

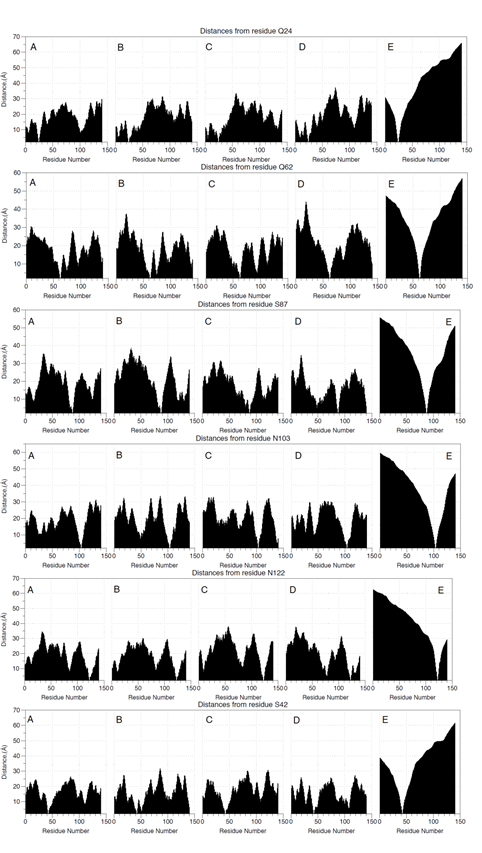

Supplement: S6 Fig — PRE-NMR α-synuclein ensemble corresponding to the publication by Allison et al., JACS 2009 [59] was obtained from Protein Ensemble Database http://pedb.vib.be. Ensemble average inter-residue distances from the residues, which were spin labeled in the PRE-NMR study. Each graph shows the average inter-residue differences between all residues in the ensemble and the spin-labelled residues in the PRE-NMR experiment. Panels A-D correspond to the four major CL-DMD clusters presented in this work, the E panels correspond to the PRE-NMR ensemble. (TIF) [file pcbi.1006859.s006.tif]

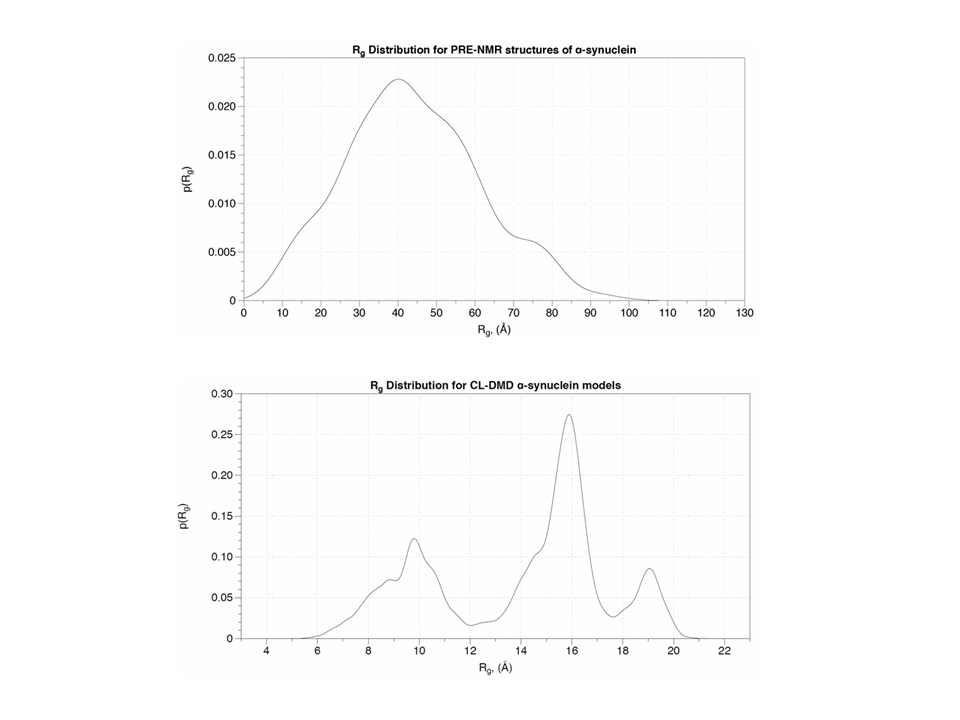

Supplement: S7 Fig — Radius of gyration (Rg) probability distributions for the ensemble of α-synuclein structures obtained by CL-DMD (combination of four major clusters) and for PRE-NMR structures downloaded from Protein Ensemble Database http://pedb.vib.be. (TIF) [file pcbi.1006859.s007.TIF]

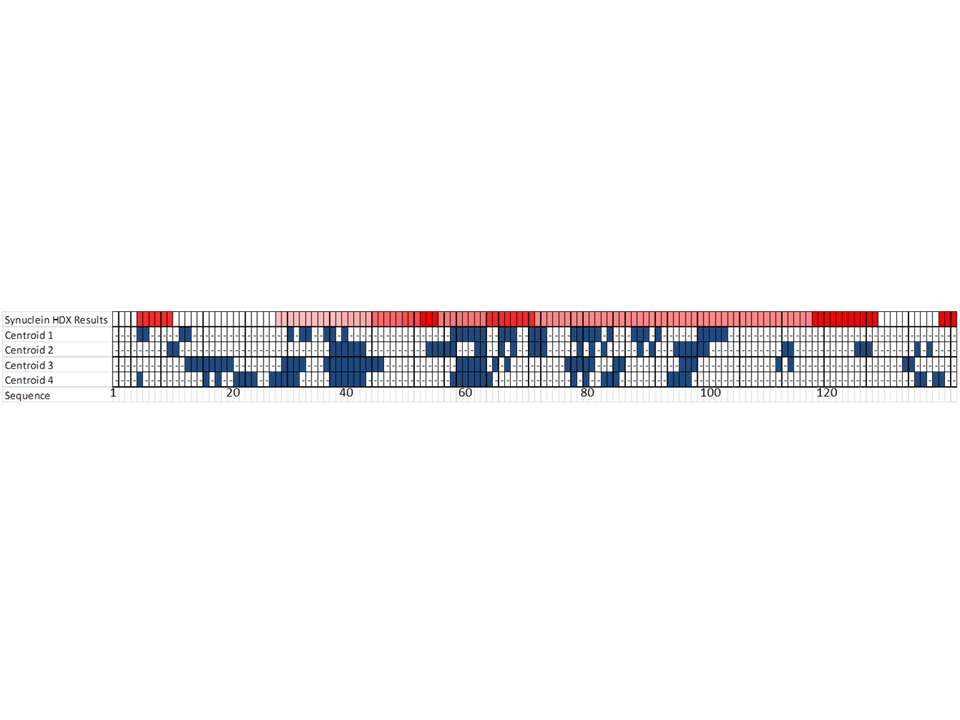

Supplement: S8 Fig — The expected protection is represented by blue boxes. The deuteration levels determined from the HDX experiments are shown using a blue-white-red gradient, with blue at 0 (protected from exchange) and red at 1 (exchanged). (TIF) [file pcbi.1006859.s008.TIF]
